# Host defense against the infection of *Klebsiella pneumoniae*: New strategy to kill the bacterium in the era of antibiotics?

**DOI:** 10.3389/fcimb.2022.1050396

**Published:** 2022-11-24

**Authors:** Zihan Liang, Yiyao Wang, Yixiang Lai, Jingyi Zhang, Lanlan Yin, Xiang Yu, Yongqin Zhou, Xinzhi Li, Yinhong Song

**Affiliations:** ^1^ Hubei Key Laboratory of Tumor Microenvironment and Immunotherapy, China Three Gorges University, Yichang, China; ^2^ Institute of Infection and Inflammation, China Three Gorges University, Yichang, China; ^3^ College of Basic Medical Science, China Three Gorges University, Yichang, China; ^4^ Affiliated Renhe Hospital of China Three Gorges University, Yichang, China

**Keywords:** *Klebsiella pneumoniae*, infection, immunity, clinical treatment, antibiotics

## Abstract

*Klebsiella pneumonia*e (*K. pneumoniae*) is a typical gram-negative iatrogenic bacterium that often causes bacteremia, pneumonia and urinary tract infection particularly among those with low immunity. Although antibiotics is the cornerstone of anti-infections, the clinical efficacy of β-lactamase and carbapenems drugs has been weakened due to the emergence of drug-resistant *K. pneumoniae*. Recent studies have demonstrated that host defense plays a critical role in killing *K. pneumoniae*. Here, we summarize our current understanding of host immunity mechanisms against *K. pneumoniae*, including mechanical barrier, innate immune cells, cellular immunity and humoral immunity, providing a theoretical basis and the new strategy for the clinical treatment of *K. pneumoniae* through improving host immunity.

## 1 Introduction

*Klebsiella pneumoniae*, a common gram-negative facultative anaerobic bacterium, widely exists not only in the natural soil and water but also in human and animal respiratory tract and intestinal tract (Paczosa and Mecsas, 2016). According to the 20-year-Antimicrobial Surveillance Program (from 1997 to 2016), *K. pneumoniae* (7.7%) ranks the third place in the most common pathogens, which is the leading cause of bloodstream infection (Diekema et al., 2019). It is easy to colonize on the surface of the human gastrointestinal and respiratory mucosa and cause pneumonia, urinary tract infections (UTIs), bacteremia and liver abscess in clinic (Paczosa and Mecsas, 2016; Choby et al., 2020). Elders, newborns and tumor patients with low immunity are generally susceptible to *Klebsiella* (Chew et al., 2017).

*Klebsiella* has several subspecies, including *K. pneumoniae subsp. pneumoniae*, *K. ozaenae subsp. ozaenae* and *K. rhinoscleromatis*. *K. pneumoniae* causes more than 95% of the common clinical cases of *Klebsiella* infection. *K. pneumoniae* strains are usually classified as classical, *hypervirulent K. pneumoniae* (*hvKp*) and multidrug resistant (MDR). Classical *K. pneumoniae* is composed of opportunistic strains often associated with hospital infection. Highly pathogenic strains, including *hvKp* are considered community-acquired bacteria that infect people of all ages, including healthy people (Wang et al., 2020). *hvKp* is a variant of *K. pneumoniae*, which is more virulent than classical *K. pneumoniae*. *hvKp* is generally resistant to the third- and fourth-generation cephalosporins and has a stronger metastatic ability. The most common metastatic sites are the eyes, lung and central nervous system, which have become the focus of clinical microbial research (Paczosa and Mecsas, 2016; Russo and Marr, 2019). Furthermore, *K. pneumoniae* is also known for its antibiotic-resistant genes, which can spread to other gram-negative bacteria. Indeed, many antibiotic-resistance genes commonly detected in multidrug-resistant organisms were first described in *K. pneumoniae* (Holt et al., 2015). Among the isolates of *K. pneumoniae* reported in the European Centre for Disease Prevention and Control, more than one-third of *K. pneumoniae* are resistant to one or more antimicrobial agents, including fluoroquinolones, the third-generation cephalosporins and aminoglycosides (Bengoechea and Sa Pessoa, 2019). The invasiveness of *K. pneumoniae* depends on its capsule, lipopolysaccharide (LPS), fimbriae and siderophores (Rodríguez-Medina et al., 2019). With the prevalence of *hvKp* and MDR, whether human will find an effective way against *K. pneumoniae* remains a mystery. Here, we focus on the relevant immune mechanism against *K. pneumoniae* to provide new clues for the clinical treatment of *K. pneumoniae* infection.

## 2 Innate Immune responses to *K. pneumoniae*

*K. pneumoniae* infection can be thought of as the outcome of interactions between *K. pneumoniae* and the host, including innate immunity and adaptive immunity. Innate immunity is the first line against *K. pneumoniae* infection, involving various barriers, innate immune cells and molecules.

### 2.1 The function of barriers

#### 2.1.1 Respiratory barriers

*K. pneumoniae* is one of the most common floras causing hospital-acquired infections and lower respiratory tract infections in the intensive care units (Saharman et al., 2020). When *K. pneumoniae* invades host, the mechanical barrier provides the immediate protection. As the interface between the host and *K. pneumoniae*, the respiratory tract and its epithelial cells play an active role as a mechanical barrier. Adult microbiota activates the defense of upper respiratory tract through interleukin (IL)-17A, while *K. pneumoniae* could overcome this obstacle to establish colonization through encapsulation (Sequeira et al., 2020). The adhesion factors with various physiological functions are present in cell wall and other structures of *K. pneumoniae* (Na et al., 2014). The colonization of *K. pneumoniae* could damage small airway epithelial cells and increase the level of tumor necrosis factor (TNF)-α in lung, and the upregulation of TNF-α could significantly exacerbate epithelial cell injury (Xu and Xu, 2005).

Additionally, the mucus ciliary layer of respiratory tract can adhere to or remove the bacteria or other particles entering the respiratory tract, while changes in the thickness, properties and cilia clearance of mucus influence the dismissal of *K. pneumoniae* by respiratory tract. Lung infection with *K. pneumoniae* could lead to massive infiltration of inflammatory cells, resulting in a progressive decrease in local defenses (Zheng et al., 2014). The outer membrane protein A of *Klebsiella pneumoniae* (KPOmpA) affects the expression of adhesion molecules and the secretion of cytokine in bronchial epithelial cells (BECs). It has been proved that KPOmpA can bind tightly to human BEC cell line BEAS-2B and primary cultures of BECs, activating the nuclear factor kappa B (NF-κB) signal pathway, thus stimulating the host defense. In addition, BECs exert internalized clearance of *K. pneumoniae* that invades the respiratory tract (Pichavant et al., 2003).

**Figure 1:**
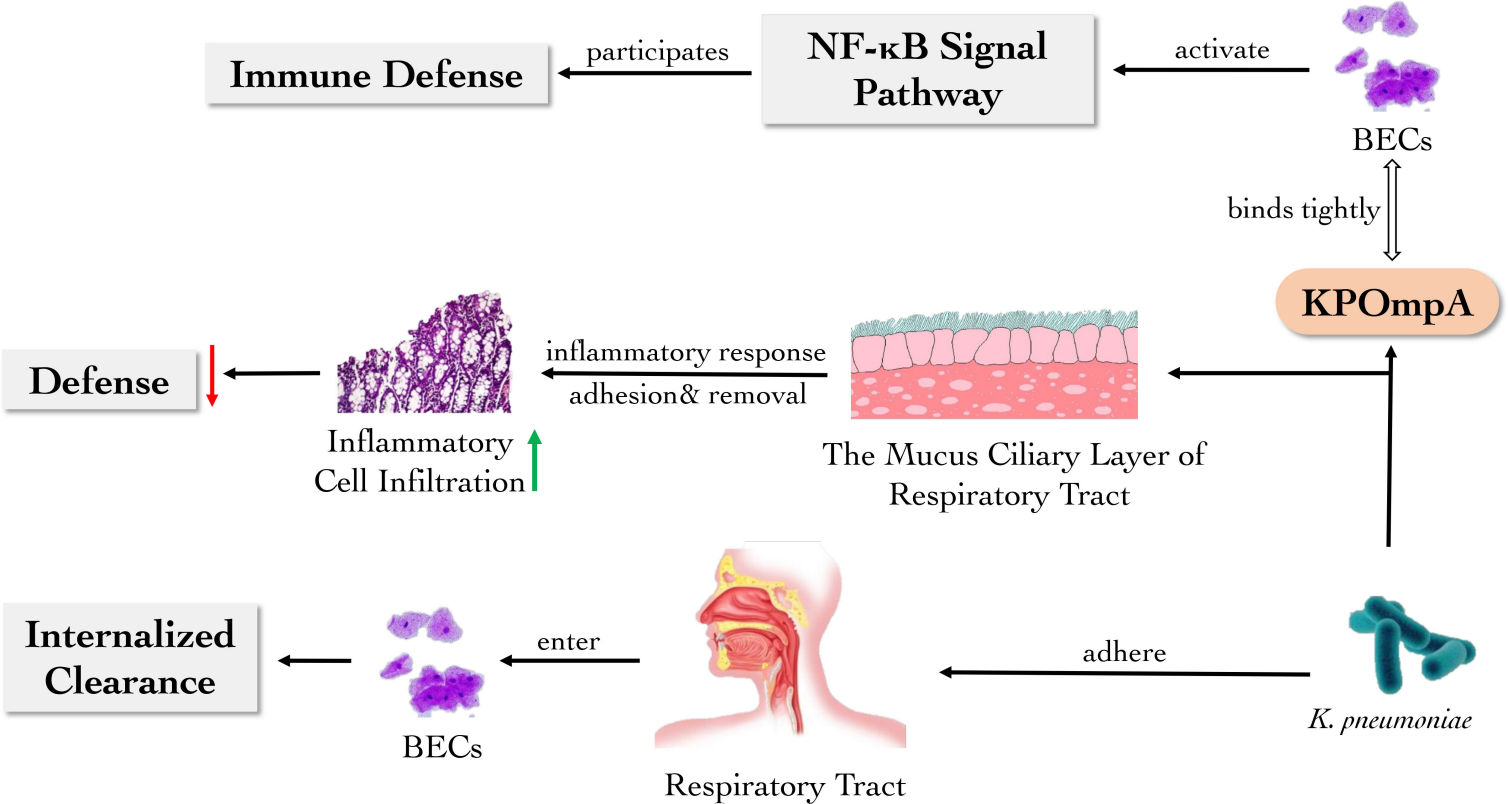
The main role of respiratory barriers against *K. pneumoniae. K. pneumoniae* can be eliminated by BECs and the mucus cilia layer of the respiratory tract. KPOmpA from *K. pneumoniae* binds tightly to BECs, thereby activating NF-κB signal pathway to participate in the host defense. However, *K. pneumoniae* and its products cause airway inflammation and weaken airway defense.

Therefore, mucus and ciliated epithelial cells in the respiratory system can effectively hinder the invasion of *Klebsiella* and eliminate it in multiple ways. However, the infection of *Klebsiella* could trigger an inflammatory response in the respiratory tract, leading to the accumulation of inflammatory cells, which can disrupt the mechanical barrier of the ciliary layer of respiratory tract and disturb the host defense (**Figure 1**).

#### 2.1.2 Urinary barriers

**Figure 2:**
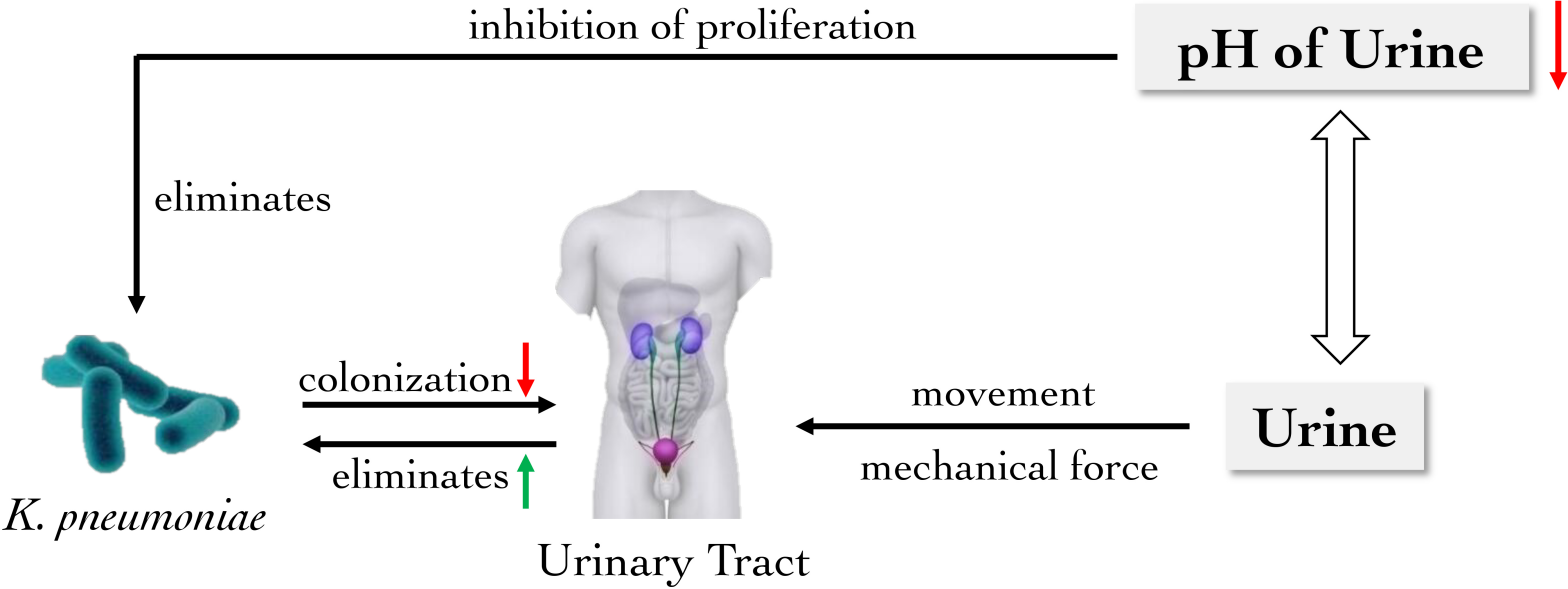
The main role of urinary barrier against *K. pneumoniae*. The mechanical force generated by the flow of urine removes *K. pneumoniae* from the urinary tract, which is also an important physical barrier to reduce bacterial colonization. Moreover, the reduction of urinary pH inhibits the proliferation of *K. pneumoniae* in the urinary tract.

Catheter-associated urinary tract infections (CAUTIs) is one of the most common nosocomial infections and complications of indwelling catheters (Maunders et al., 2022). *K. pneumoniae* is prone to UTIs through catheters, accounting for 2-6% of hospital UTIs (Li et al., 2014; Maunders et al., 2022). However, the mechanical force created by the flow of urine can remove pathogens normally, which acts as an essential barrier for the colonization of bacteria. Furthermore, the pH value of urine is a critical factor in the colonization and proliferation of pathogenic bacteria in the urinary tract, and the alteration of pH value may play an important role in the treatment and prevention of *Klebsiella* on UTIs (Yang et al., 2014; Wasfi et al., 2020). Meanwhile, the bladder smooth muscle activity could significantly increase the positive rate of *Klebsiella* in urine, which allows for flushing *Klebsiella in vivo* (Burnett et al., 2021). It is known that *K. pneumoniae* may adhere to the host cell surface with the help of various adhesion factors such as the *K. pneumoniae* MrkD adhesin, colonizing the host and causing infections (Jagnow and Clegg, 2003; Li et al., 2009). Fortunately mechanical forces such as urine activity, bladder contraction, and the alteration of pH value in urine are capable of weakening the colonization of *K. pneumoniae* (**Figure 2**).

#### 2.1.3 Digestive barriers

Studies have shown that the main anti-*Klebsiella* effect of digestive system comes from gut microbiota. The gut microbiota consists of diverse bacterial communities that perform various functions and influence the host’s overall health, including nutrient metabolism, immune system regulation and natural defense against infection (Al Bander et al., 2020). During the *K. pneumoniae* infection, there is a complex interaction between the host and gut microbiota.

Researches have shown that in the early stage of *K. pneumoniae* infection, the richness and composition of gut microbiota changes, especially the numbers of *Lactobacillus reuteri* and *Bifidobacterium pseudolongum* decrease significantly (Wu et al., 2020; Wolff et al., 2021). Among the gut microbiota, *Bacteroidetes* can strengthen the intestinal immune barrier through IL-36 and macrophages to prevent the colonization and transmission of *K. pneumoniae* (Sequeira et al., 2020).

Short-chain fatty acids (SCFA), fermentation products of intestinal flora, including acetic acid, butyric acid and propionic acid, play a pivotal role in resisting the colonization and inflammation of *K. pneumoniae*. Vornhagen et al. observed that SCFA could directly inhibit bacterial growth through intracellular acidification in a dose-dependent manner. SCFA also reduces epithelial oxygenation and stimulate the expression of antimicrobial peptides in the gut microbiota, thus weakening pathogen colonization. Further, SCFA affects intestinal homeostasis to induce gut microbiota to produce metabolites, thereby decreasing the fitness of *K. pneumoniae* lacking functional plasmid encoding tellurite TeO3-2-resistance (Ter) operons in the intestinal tract (Vornhagen et al., 2021). Another research also showed that the G protein-coupled receptor 43 (GPR43) combined with acetate could upregulate the activity of neutrophils and alveolar macrophages, which reduce the number of bacteria in the airway in the early stage of infection, and promote inflammation regression to reduce lung injury in the late stage of infection. These results indicate that GPR43 plays a significant role in the “gut–lung axis” as a sensor of the host gut microbiota activity. Increasing SCFA will probably be a new way to promote inflammation resolution in clinical practice (Galvão et al., 2018). Aside from that, butyrate and tryptophan decomposition metabolites are able to enhance gut integrity and stimulate innate lymphoid cells group 3 (ILC3) to produce IL-22. Gut microbiota also could reduce intestinal permeability and increase the epithelial defense mechanism to form a mucosal barrier. Therefore, they maintain the stability of the intestinal environment (Shi et al., 2017). In the case of liver abscess induced by *K. pneumoniae*, relevant studies have discovered that antibiotic treatment before *K. pneumoniae* infection weakens the protective effect of intestinal flora in mice. Surprisingly, after fecal transplantation, the concentrations of chemokine (C-X-C motif) ligand 1 protein (CXCL1), TNF-α, monocyte chemoattractant protein-1 (MCP-1), IL-1β, IL-6 and IL-17 in mice serum were recovered and liver injury was alleviated (Zheng et al., 2021).

**Figure 3:**
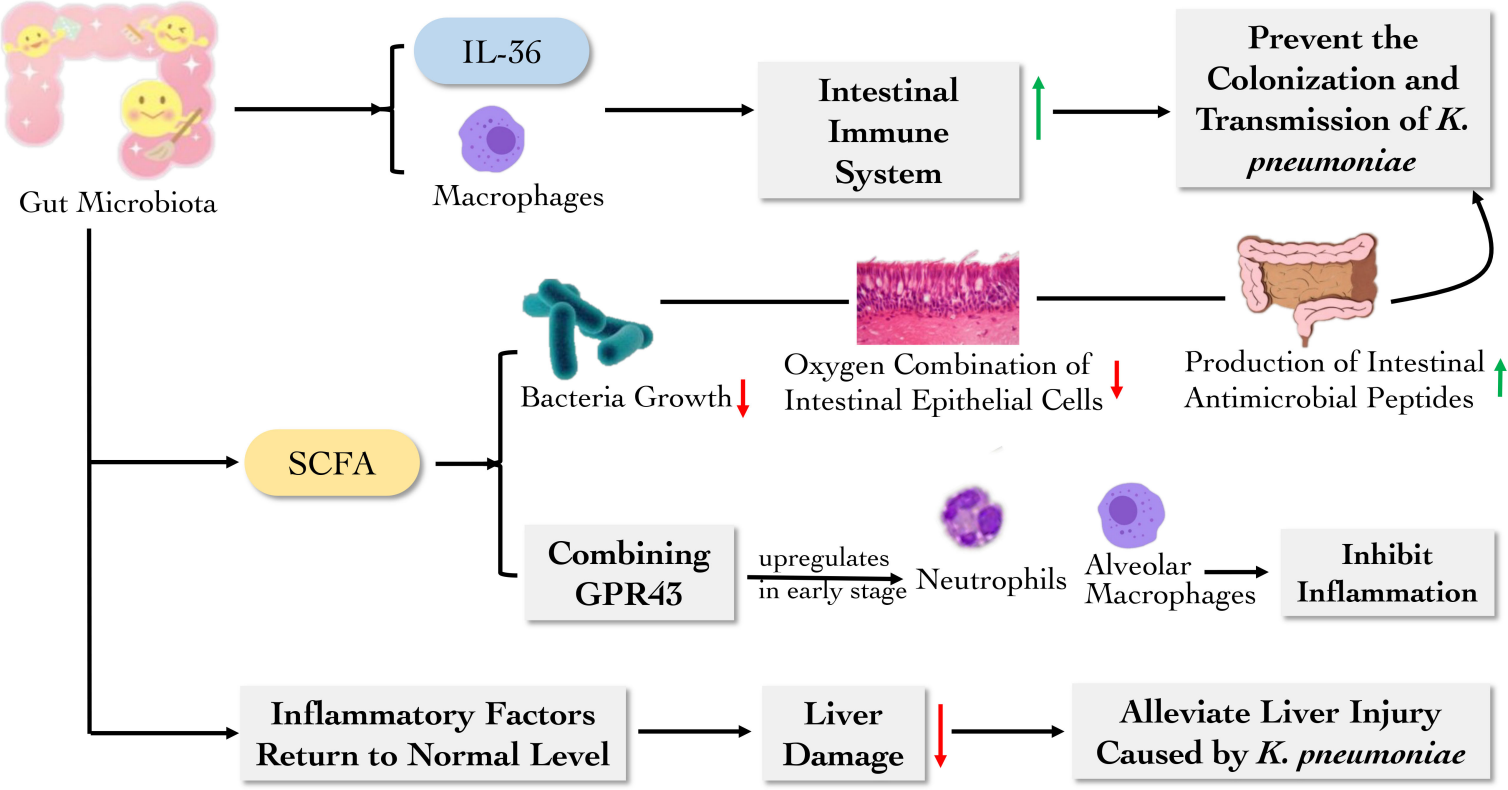
The main role of digestive barrier against *K. pneumoniae*. The gut microbiota and their metabolites can reduce the colonization and transmission of *K. pneumoniae* and inhibit inflammation by regulating the activity of immune cells and inflammatory factors.

In a word, gut microbiota and its metabolites is essential in *K. pneumoniae* colonization and inflammatory response (**Figure 3**). Administration of exogenous SCFA could be sufficient to reduce fitness of *K. pneumoniae*. However, whether other substances also have impacts and how these microorganisms and metabolites interact with the host remains need to be further explored.

### 2.2 The function of innate immune cells

#### 2.2.1 Dendritic cells

**Figure 4:**
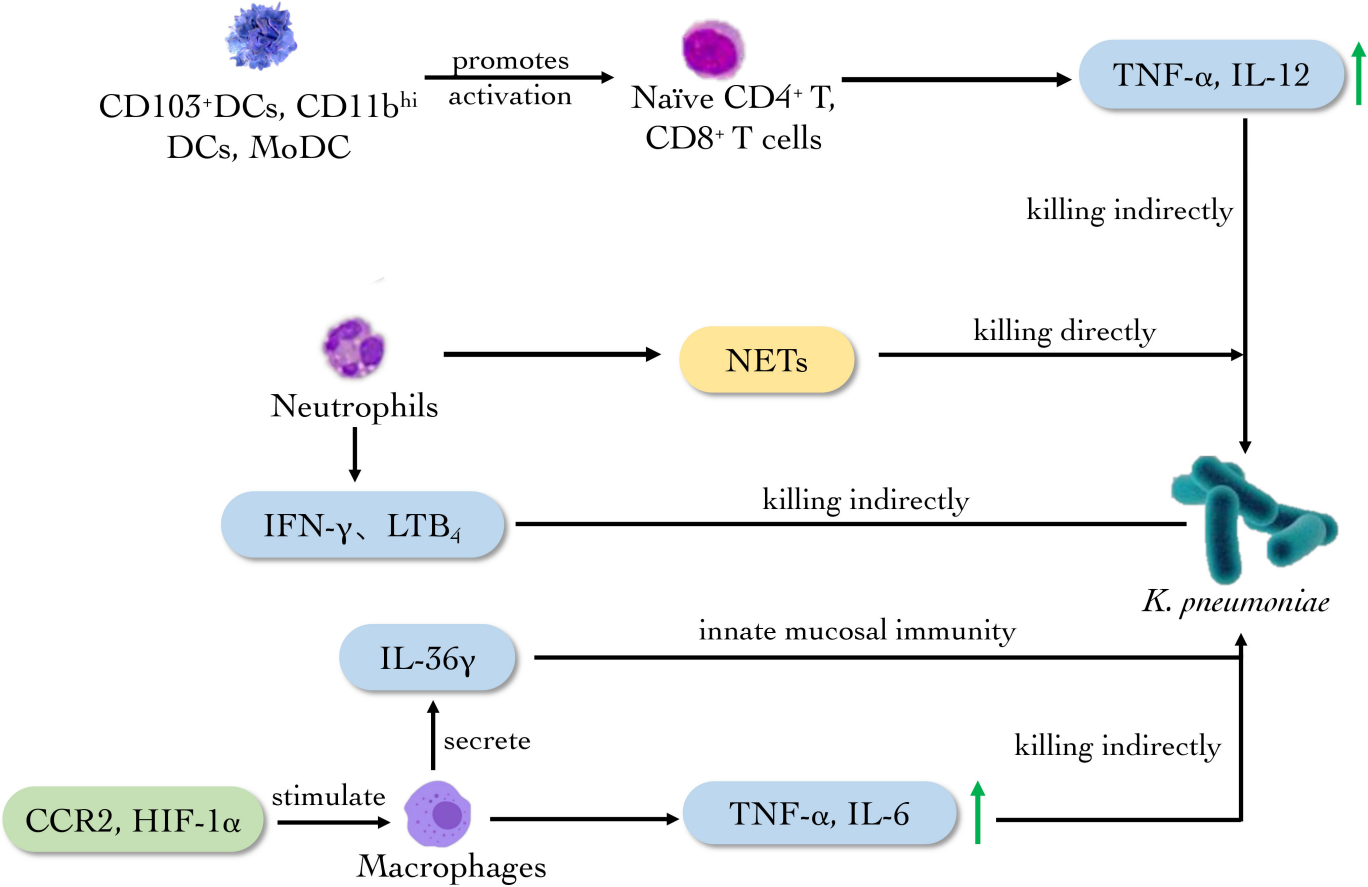
The main role of innate immune cells against *K. pneumoniae*. Neutrophils produce NETs to eliminate *K. pneumoniae* directly. Meanwhile, CD103^+^DCs, CD11b^hi^ and MODCs promote the activation of naïve T cells. CCR2 and HIF-1a activate macrophages to secrete cytokines such as TNF-α, IL-6 and IL-36γ to eradicate the bacterium.

Dendritic cells (DCs) of the lung are situated in close proximity to alveolar epithelium and resident alveolar macrophages, playing a specific role as antigen-presenting cells (APCs) (Von Wulffen et al., 2007). There are several subtypes of DCs. Plasmacytoid DCs (pDCs) can produce interferon (IFN)-α and sense the damaged skin to heal wounds. CD103^+^ DCs, CD11b^hi^ DCs and monocyte-derived DCs (MoDCs) can act as migratory DCs to promote the activation of naïve CD4^+^ and CD8^+^ T cells in lymph nodes (Hackstein et al., 2012; Plantinga et al., 2013). Hackstein et al. discovered a rapid increase of activated CD103^+^ DC, CD11b^+^ DC and MoDC within 48 h post infection of *K. pneumoniae.* The *K. pneumoniae*-infected animals showed that in respiratory DC subpopulations there were elevated IFN-α in pDC, elevated IFN-γ, IL-4 and IL-13 in CD103^+^ DC and IL-19 and IL-12p35 in CD11b^+^ DC subsets in comparison to CD11c^+^ MHC-class II^low^ cells indicating distinct functional roles. CD103^+^ DC and CD11b^+^ DC subsets represented the most potent naïve CD4^+^ T helper cell activators in the infection model of *K. pneumoniae* (Hackstein et al., 2013) (**Figure 4**). Therefore, the novel insight into the activation of respiratory DC subsets during *K. pneumonia* infection is provided.

#### 2.2.2 Macrophages

Pulmonary macrophages are derived from monocytes, which mainly stimulate other immune cells acting as APCs and secreting immune molecules. The latest experiments showed that capsular polysaccharide (CPS) derived from carbapenem-resistant *K. pneumoniae KN2* serotype can stimulate J774A.1 mouse macrophage to release TNF-α and IL-6 *in vitro*. The CPS also exerts an immune response through TLR4 in human embryonic kidney-293 (HEK-293) cells (Lee et al., 2022). Melissa and Kovach observed that the clearance rate of *K. pneumoniae* in IL-36γ-deficient mice was decreased and the mortality of the mouse was increased, which confirmed that IL-36γ is related to the anti-*Klebsiella* effect. Further, it is proved that pulmonary macrophages secreted IL-36γ in a non-Golgi-dependent manner, playing a critical role in innate mucosal immunity of lung (Kovach et al., 2016; Kovach et al., 2017). The chemokine-mediated transportation of mononuclear phagocytes also is essential in the defense against bacterial pneumonia. In the *K. pneumoniae*-infected mouse model, the deletion of chemotactic cytokines receptor 2 (CCR2) could reduce all the monocyte phagocyte subsets and change the phenotype of pulmonary macrophages, reducing the amount of M1 macrophages and TNF in lung (Chen et al., 2013). Myeloid but not neutrophil-specific hypoxia-inducible factor (HIF)-1α-deficient mice increased bacterial loads in the lungs and distant organs after infection of *K. pneumoniae* as compared to control mice, pointing to a role of HIF-1α in macrophages. What’s more, alveolar and lung interstitial macrophages from myeloid-specific HIF-1α-deficient mice produced a lower level of immunity, suggesting the importance of HIF-1α expressed in lung macrophages in protective innate immunity during pneumonia caused by *K. pneumoniae* (Otto et al., 2021).

Collectively, the researches above indicate that pulmonary macrophage is essential in innate immunity by secreting cytokine such as IL-36, TNF-α and IL-6 after *K. pneumoniae* infection, while CCR2 and HIF-1α play an auxiliary role in the anti-*K. pneumoniae* activity of macrophages (**Figure 4**). These discoveries provide new ideas for the clinical treatment of pneumonia caused by *Klebsiella* infection.

#### 2.2.3 Neutrophils

Neutrophils are the first line against a variety of infectious pathogens. Neutrophils could kill pathogens by phagocytosis and neutrophil extracellular traps (NETs). NETs is one of the primary defensive mechanisms of neutrophils against *carbapenemase resistant hypervirulent K. pneumoniae* (*CR-hvKp*). Through the scanning electron microscope test, Jin et al. found that the NETs in type 2 diabetes patients had lost its smooth and regular shape, which may lead to the defection of congenital immune response for the patients against *CR-hvKp*. The study confirmed the direct killing effect of NETs to *CR-hvKp* (Jin et al., 2020). More recently researchers compared the concentration of cytokines in regular diet group of mice and high-fat diet group infected with *K. pneumoniae*, discovering that the concentrations of IL-1β, IL-6, IL-17, IFN- γ, CXCL2 and TNF-α were much lower in the high-fat diet group, meanwhile, the number of neutrophils was reduced, and the functions including the phagocytosis, killing ability and production of the reactive oxygen intermediates (ROI) were impaired significantly, which proved the critical role of neutrophils in anti-*K. pneumoniae* effect (Mancuso et al., 2022).

Further studies found that the expression of CXCL5 in IL-17-deficient epithelium decreased, while intranasal injection of recombinant CXCL5 in mice could restore neutrophils’ recruitment and bacterial clearance (Chen et al., 2016). It was also discovered that the number of neutrophils decreased and the production of leukotriene B_4_ (LTB_4_), reactive oxygen species (ROS) and reactive nitrogen species (RNS) decreased in CXCL1-/- mice infected with *K. pneumoniae* (Batra et al., 2012). Meanwhile, subsequent experiments on depleted neutrophils showed that neutrophils were the main source of LTB_4_ in the lungs after infection (Batra et al., 2012). The above researches reveal the important role of CXCL1 in the expression of ROS and RNS produced by neutrophils, the regulation of host immunity to *K. pneumoniae* infection, and the curative effect of LTB_4_ on the recruitment of neutrophils. In addition, more studies have shown that IL-33 can enhance host defense during bacterial pneumonia through the combined function of neutrophils and inflammatory monocytes (Ramirez-Moral et al., 2021).

Evidences *in vitro* and *in vivo* above indicate that neutrophils play an anti-*K. pneumonia’s* role mainly through NETs and secreting corresponding molecules such as LTB_4_, ROS and RNS (**Figure 4**). What’s more, the administration of CXCL5 and LTB_4_ can restore the activity of neutrophils, providing a new direction for the clinical treatment of *K. pneumoniae*.

#### 2.2.4 Innate Lymphoid Cells

**Figure 5:**
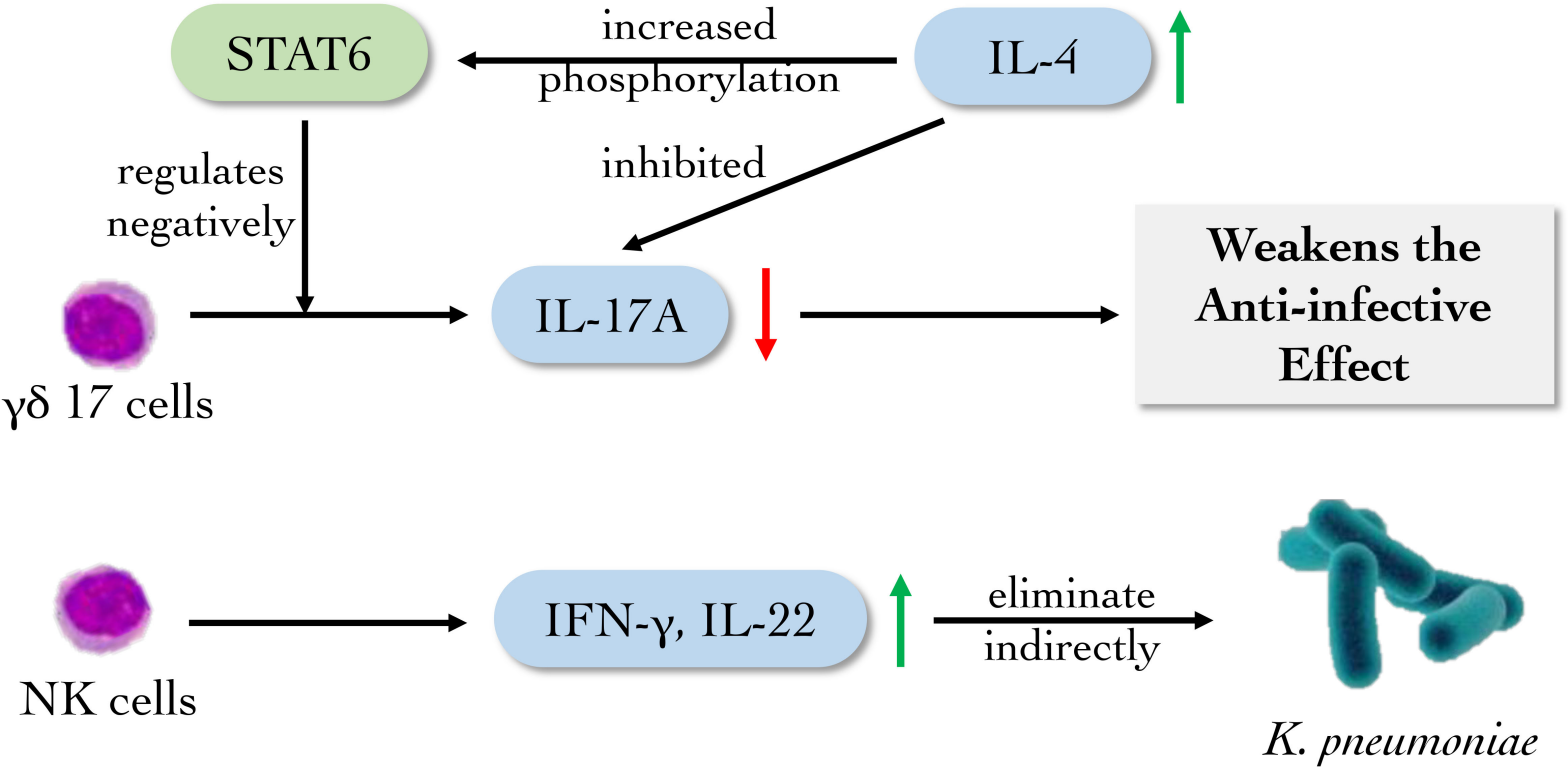
The main role of innate lymphoid cells against *K. pneumoniae*. NK cells and its subsets mainly eliminate *K. pneumoniae* indirectly by producing cytokines. γδ17 cells facilitate the eradication of *K. pneumoniae* by secreting IL17A, which can be suppressed by high level of IL-4.

All T lymphocytes including T_H_17 and γδT subsets participating in innate immunity derive from common lymphoid progenitor in bone marrow, differentiate and mature in thymus. γδ17 cell is a subset of γδT cells which produce large quantities of IL-17A in the presence of IL-23 and IL-1β. γδ17 cell expresses the type I IL-4R. And IL-4 signaling increases STAT6 phosphorylation in γδT cells. Whereas IL-4 inhibits the production of IL-17A by γδ17 cell. *K. pneumoniae* infection of STAT6 knockout mice shows a higher amount of γδ17 cell compared to that of wild-type mice, demonstrating that STAT6 signaling negatively regulates γδ17 cell that play a front-line role in mucosal immunity against *K. pneumoniae* (Bloodworth et al., 2016). Recently, Mackel et al. evaluated the role of T cells in protection against classical *K. pneumoniae* reinfection and demonstrated that mice lacking T cells were unable to establish a protective response. However, mice individually deficient in either of the major T cell subsets, γδ or αβ (classical T cells), effectively mounted a protective response, indicating either subset alone was sufficient to mediate protection against the reinfection of *K. pneumoniae* (Mackel et al., 2022) (**Figure 5**). The researches above demonstrate the imperative contribution of innate T cells to protective immunity against classical *K. pneumoniae* and will guide further inquiries into host effector responses required to control *K. pneumoniae* infection.

Nature killer (NK) cells, which belong to innate lymphoid cells (ILCs), are found to recognize and eliminate “altered self” as cytotoxic lymphocytes, which also take part as the source of early inflammatory cytokines in the innate immune system (Pallmer and Oxenius, 2016; Myers and Miller, 2021). After activation, they secrete perforin and TNF to kill “allosome” substances nonspecifically. It was found that after infection with *K. pneumoniae*, the survival rate of IL-22^-/-^ mouse was lowered while the survival rate of Rag2^-/-^ mouse had no significant changes compared with wild-type mouse. Simultaneously, Rag2^-/-^Il2rg^-/-^ mice failed to produce IL-22. NK cells and T cells may produce IL-22 and have conventional host defense against *K. pneumoniae*, which were confirmed with Rag2^-/-^Il2rg^-/-^ C57BL/6 mice (Xu et al., 2014). Type I IFN receptor (*Ifnar*) 1-deficient mice infected with *K. pneumoniae* failed to activate NK cells to produce IFN-γ, which caused the weakening of NK cell killing effect. Meanwhile, exogenous IFN-γ can recover the level of IFN-γ in *Ifnar1* ^fl/fl^ (Ifnar1^tm1Uka^)-CD11c^Cre^, *Ifnar1* ^fl/fl^-LysM^Cre^ and *Ifnar1* ^fl/fl^-MRP8^Cre^ mice on C57BL/6 background (Ivin et al., 2017). These data identify NK cell-intrinsic type I IFN signaling as essential driver of *K. pneumoniae* clearance, and reveal a specific target for future therapeutic exploitations (**Figure 5**).

### 2.3 The function of innate immune molecules

#### 2.3.1 TLRs

Innate immunity depends on signals produced by pattern recognition receptors (PRRs). Toll-like receptors (TLRs) is the earliest PRRs, which can recognize pathogen-associated molecular patterns (PAMPs) or damage-associated molecular patterns (DAMPs) in microorganisms (O’neill et al., 2013). When the ligand binds to TLRs, myeloid differentiation factor88 (MyD88) and Toll/IL-1R (TIR) domain-containing adaptor protein (TIRAP) are recruited into the TLR complex to activate MAPK and NF-κB signal pathways to produce cytokines and chemokines. This cascade reaction is called MyD88-dependent pathway. In the lung against *K. pneumoniae* TIRAP is a critical mediator of antibacterial defense (Jeyaseelan et al., 2006). Besides, the activation of TLRs also recruits other adapter proteins, such as TIR domain-containing adaptor-inducing IFN-β (TRIF) and TRIF-related adaptor molecule (TRAM). This pathway activates NF-κB and type I IFN, which is called TRIF-dependent pathway. Both TRIF-dependent and MyD88-dependent signaling contributes to host defense against pulmonary *Klebsiella* infection (Cai et al., 2009). The TLR-mediated innate immune responses control bacterial growth at the infection site, thus minimizing bacterial transmission. Currently, 12 TLRs from mice and 10 TLRs from human have been identified (Baral et al., 2014).

Among TLRs, TLR2 and TLR4 play important roles in *K. pneumoniae* infection. TLR2 transmits signal mainly by forming heterodimers with TLR1 or TLR6 to resist external pathogens. Meanwhile, TLR4 could induce host defense against gram-negative bacterial pulmonary infection by sensing bacterial LPS (Baral et al., 2014). Compared with wild type (WT) mice, Jeon et al. found that the survival time of TLR4 knock-out (KO) and TLR2/4 double KO (DKO) mice infected with 5×10^3^ CFU *K. pneumoniae* was significantly shortened. The mRNA levels of TNF-α, MCP-1 and inducible nitric oxide synthase (iNOS) in TLR2/4 DKO mice were substantially lower than those in the WT group, indicating that TLR2 and TLR4 play a synergistic role in innate immune response during *K. pneumoniae* infection (Jeon et al., 2017).

Meanwhile, by analyzing the gene expression profiles in the lung of C57BL/6 mice (resistant to bacterial transmission), 129/SVJ mice (susceptible), C3H/HeJ mice (susceptible and TLR4 signal deficient) and their respective control strains C3H/HeN mice (moderately resistant), it was found that the most significant number of TLR4-dependent induced genes were expressed in C57BL/6 and C3H/HeN mice after infection with *K. pneumoniae*. These genes include cytokines and chemokine genes needed for neutrophil activation or recruitment, growth factor receptors, MyD88 and adhesion molecules. The results indicated that in the early stage of infection, the TLR4 signal controlled the expression of most genes in lung to cope with gram-negative bacterial infection (Schurr et al., 2005).

At the same time a variety of TLRs expressed on DCs, such as TLR9, can trigger the cascade signal response of proinflammatory cytokines, leading to the production of TNF-α, IL-12 and other proinflammatory cytokines in large quantities to resist the invasion of *K. pneumoniae* (Bhan et al., 2007; Von Wulffen et al., 2007). Interestingly, though the initiation of most TLRs depends on MyD88, Adam et al. discovered that the inflammatory response induced by *K. pneumoniae* does not depend on MyD88 in lung epithelial cells and platelets (De Stoppelaar et al., 2015; Anas et al., 2017).

**Figure 6:**
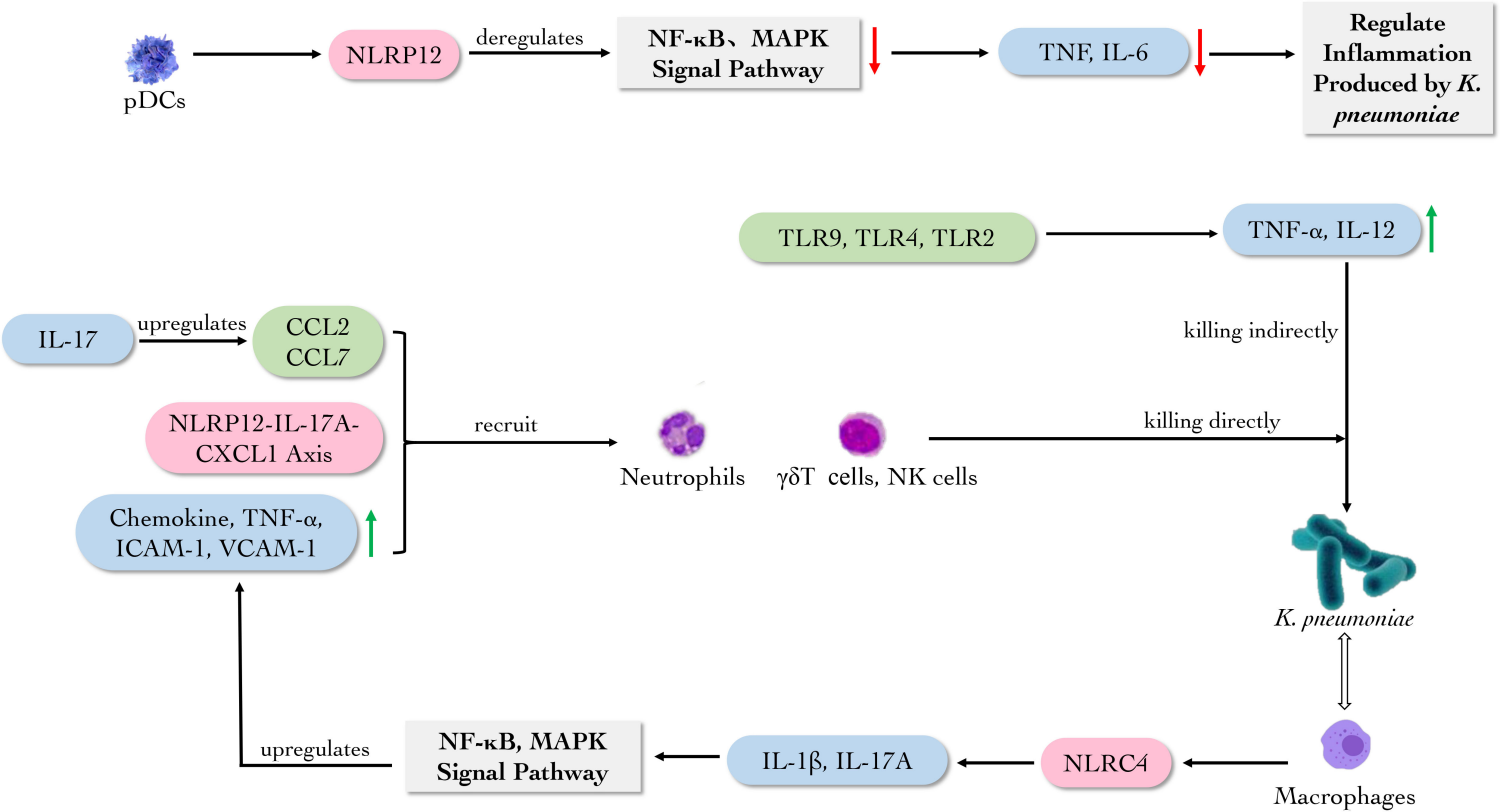
The main role of immune molecules against *K. pneumoniae*. pDCs secrete NLRP12 participating NF-кB and MAPK signal pathways to regulate the inflammation caused by *K. pneumoniae*. While TLR2, TLR4 and TLR9 promote the production of TNF-α and IL-12. Macrophage-derived NLRC4 induces the production of IL-1 and IL-17A in lung, activating NF-κB and MAPK signal pathways to upregulate the production of chemokine, TNF-α, ICAM-1 and VCAM-1, which can recruit neutrophils. Meanwhile, NLRP12-IL-17A-CXCL1 axis, CCL2 and CCL7 recruit neutrophils, γδT cells and NK cells to eliminate *K. pneumoniae*.

Collectively, TLR2 and TLR4 signaling could improve the levels of TNF-α, MCP-1, iNOS and other proinflammatory cytokines to indirectly eliminate the bacteria during the early stage of *K. pneumoniae* infection (**Figure 6**).

#### 2.3.2 NLRs

Nucleotide-binding oligomerization domain (NOD)-like receptors (NLRs) are classical pattern recognition receptors highly expressed in first-reactive cells such as neutrophils, macrophages and DC cells. They can regulate various signal pathways, including MyD88 and TLRs containing adaptor molecular 1-dependent pathways. Besides these regulatory effects, some NLRs also are assembled into polymer protein complexes called inflammasome (Ravi Kumar et al., 2018; Ghimire et al., 2020).

Nucleotide-binding oligomerization domain, leucine- rich repeat and pyrin domain-containing (NLRP) is one category of NLRs. Studies have shown that NLRP6 and NLRP12 can act as a negative regulator of the NF-κB and mitogen-activated protein kinase (MAPK) signal pathways to attenuate intestinal inflammation during the infection of *K. pneumoniae* (Ghimire et al., 2020). NLRP6 and the adaptor protein, apoptosis-associated speck-like protein (ASC) -mediated inflammasome activation are thought to shape the composition of the commensal gut microbiota, controlling the gut microbiota and the immune response to systemic and intestinal infections (Elinav et al., 2011). However, the finding was challenged by later research (Mamantopoulos et al., 2017). It is meaningful that during the infection of *K. pneumoniae*, *NLRP6* gene-deficient mice show the low levels of neutrophil recruitment, CXC chemokine and granulocyte factor (Cai et al., 2021).

Furthermore, bone marrow-derived DCs (BMDCs) lacking NLRP12 could induce the production of TNF-α and IL-6 (Allen et al., 2013; Tuladhar and Kanneganti, 2020). It was found that intratracheal injection of IL-17A^+^ CD4 T cells or CXCL1^+^ macrophages could prolong the survival of *Nlrp12* ^-/-^ mice and recruit neutrophils to eliminate *K. pneumoniae*. It was revealed that the NLRP12-IL-17A-CXCL1 axis *in vivo* could play a vital role in removing extracellular bacteria by recruiting neutrophils (Cai et al., 2016). And another study found that IL-17 can upregulate the expression of CCR2 ligands CCL2 and CCL7, promoting the recruitment of neutrophils and enhancing the anti-bacterial activity in C57BL/6 mice (Xiong et al., 2015).

NLRC4, another inflammasome, is also essential for the clearance of *K. pneumoniae* and neutrophil-mediated lung inflammation (Xiong et al., 2015; Xiong et al., 2016). Macrophage-derived NLRC4 can induce the production of IL-1 and IL-17A from NK cells and γδT cells in lung, activating NF-κB and MAPK signal pathways to regulate the production of neutrophil chemokine, TNF-α, the expression of intercellular cell adhesion molecule-1 (ICAM-1) and vascular cell adhesion molecule-1 (VCAM-1) in lung homogenates, which could recruit neutrophils and hinder the colonization of *K. pneumoniae* (Cai et al., 2012).

These evidences revealed that NLRP6 and NLRP12 could act as negative regulators of NF-κB and MAPK signal pathways to deregulate the inflammation caused by *K. pneumoniae.* Meanwhile, NLRs prevent the colonization of *K. pneumoniae* by recruiting neutrophils (**Figure 6**).

## 3 Adaptive Immune Response to *K. pneumoniae*

It is known that innate immunity and adaptive immunity are equally crucial in resisting pathogen invasion, the adaptive immune response is slower and more specific. Another prominent feature for adaptive immunity is the production of immunologic memory which is activated rapidly during reinfection, resulting in protective response (Bonilla and Oettgen, 2010; Opoku-Temeng et al., 2022). Adaptive immunity includes T cell-mediated cellular response and B cell-mediated humoral response (Bonilla and Oettgen, 2010).

### 3.1 T Cell-mediated Immune Response

As *K. pneumoniae* is a typical extracellular bacterium, the effect of cellular immunity on *K. pneumoniae* is relatively limited (Opoku-Temeng et al., 2022). Lee et al. found that T-helper (T_H_) lymphocytes played a prominent role in the defense of *K. pneumoniae* through secreting cytokines such as IL-17 and IFN-γ (Lee et al., 2015). It was found that resident memory T cells (T_RM_) also played an anti-*Klebsiella* role through lung mucosal immunity. Vesely et al. discovered that the lung long-lived CD4 T_RM_ cells derived from T_H_17 cells could rapidly release IFN-γ or release IL-4 later to better control infections of CR-hvKp or contribute to the pathology associated with the hypersensitivity (Amezcua Vesely et al., 2019). Meanwhile, the newly studied vaccine can drive lung T_RM_ cells to provide immunity against *Klebsiella via* fibroblast IL-17R signaling (Iwanaga et al., 2021).

### 3.2 Humoral Immune Response

It is different from cellular immunity, humoral immunity exerts an enormous function in host defense against *K. pneumoniae* infection. Banerjee et al. had isolated cross-reactive anti-CPS antibodies poly-immunoglobulin G (poly-IgG) from the plasma of patients infected with *carbapenem-resistant K. pneumoniae* (*CR-Kp*) strain sequence type 258 (ST258), which indicated poly-IgG could promote the phagocytic function to different serotype *CR-Kp* strains. Still, the protective efficacy was reversed when CPS-specific antibodies (Abs) were depleted (Banerjee et al., 2021). Diago et al. isolated K1-CPS-specific IgG Abs and found that in mouse liver, monoclonal antibodies (mAbs) 4C5 and 19A10 reduced the transmission of *CR-hvKp* with *in vivo* microscope (Diago-Navarro et al., 2017). Subsequently, two anti-CPS IgG mAbs 17H12 and 8F12 were obtained by Diago et al. from the mouse infection model. The two mAbs can promote extracellular processes to kill *CR-Kp*, including the enhancement of biofilm inhibition, the deposition of complement and NETs, reducing bacterial transmission to organs (Diago-Navarro et al., 2018). Motley and his fellows also isolated two anti-CPS mAbs, murine IgG3 (mIgG3) and murine IgG1 (mIgG1), revealing that mIgG3 had better complement-mediated serum bactericidal activity than mIgG1, and promoting neutrophil-mediated killing at a concentration below mIgG1 through enzyme-linked immunosorbent assay and flow cytometry. In contrast, mIgG1 had better activity in enhancing the phagocytosis of macrophages (Motley et al., 2020).

Kobayashi et al. tested CPS-specific rabbit Abs and found that CPS2-specific Abs can promote phagocytosis and the pernicious effect of human neutrophils to ST258 (Kobayashi et al., 2018). Observing the interaction of African green monkey complement and antibodies with hyper mucoviscosity (HMV) or non-HMV *K. pneumoniae*, the results demonstrate that interaction of cellular and humoral immune elements plays a role in the *in vitro* killing of *K. pneumoniae*, particularly HMV isolates. However, low levels of IgG2 titers may lead to a diminished sterilization effect (Soto et al., 2016). The increased prevalence of *K. pneumoniae* LPS O2 serotype strains in all significant drug resistance groups correlates with a paucity of anti-O2 antibodies in human B cell repertoires. It has been identified that human mAbs to O antigen, including a rare anti-O2 specific antibody, is highly protective in mouse infection models, even against heavily encapsulated strains (Pennini et al., 2017).

Furthermore, an isolated antibody B39 targeting conserved epitope binds to *K. pneumoniae* LPS O1 and O1/O2 antigens could promote the conditioning phagocytosis of human macrophages and the clearance of macrophage-associated bacteria when evaluating them by high-volume image (Berry et al., 2022). At the same time, Lee. et al. found that *K. pneumoniae* EV vaccination conferred protection against *K. pneumoniae* infection by inducing EV-reactive Abs and IFN-γ^+^ T-cell responses. It indicates that *K. pneumoniae* EV vaccination depends on both humoral and cellular immunity (Lee et al., 2015). Similarly, stable artificial bacterial bionic vesicles (BBVs) were successfully induced and efficiently taken up by DCs to stimulate DCs’ maturation. Therefore, as a *K. pneumoniae* vaccine, BBVs could induce bacterial-specific humoral and cellular immune responses to reduce lung inflammation and its bacterial load (Li et al., 2021).

**Figure 7:**
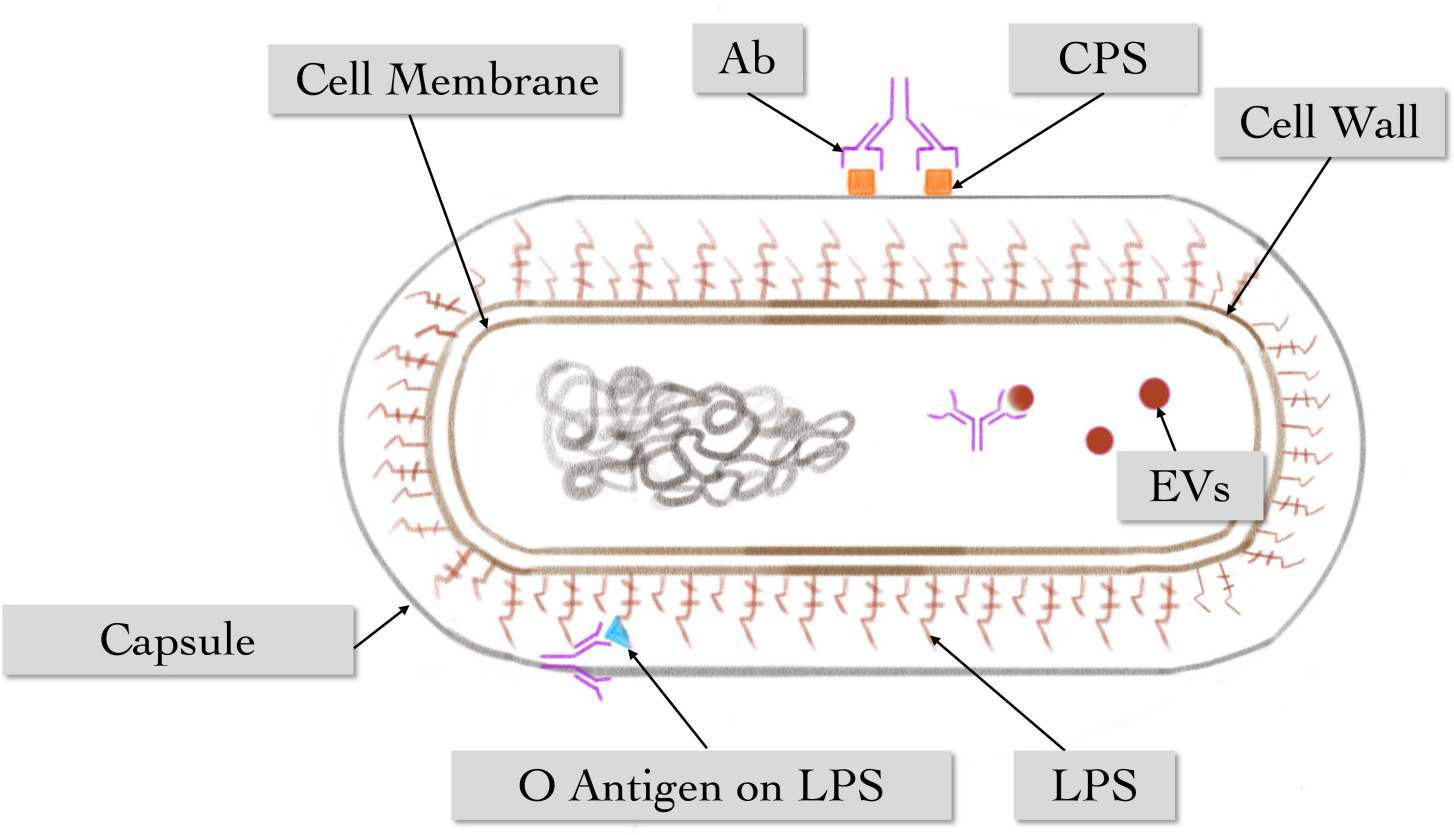
The main role of humoral immune response against *K. pneumoniae*. Multiple antibodies can act on different antigenic epitopes of *K. pneumoniae*, including vesicles, O-antigen on LPS, and antigen on CPS.

Collectively, these studies strongly suggest the critical role of humoral immunity, which is underestimated in clinical applications in terms of antibiotic therapy. Due to the protective potential of anti-CPS, *K. pneumoniae* CPS is a popular target for immune prevention and/or treatment, and the O antigen of LPS and EVs are also viable targets (**Figure 7**). In addition, antibody-based clinical treatment strategies may have the capacity to address antibiotic-refractory bacteria in the future.

## 4 Discussion and conclusion

Over the past decade, *K. pneumoniae* has emerged as a significant clinical and public health threat due to the increasing prevalence of healthcare-associated infections caused by multidrug-resistant strains that produce extended-spectrum β-lactamases and/or carbapenemases. Here, the immune mechanisms associated with the resistance to *K. pneumoniae*, including innate immunity, cellular immunity and humoral immunity have been reviewed (**Table 1**). The mechanical barriers play a preliminary role in anti-colonization, while humoral immunity antibodies can recognize different antigenic epitopes of *K. pneumoniae* to promote the elimination of the pathogen. At the same time, multiple immune cells are activated and able to secrete relevant immune factors to aggregate and destroy the infected cells when the organism is infected with *Klebsiella*, demonstrating the diversity and effectiveness of immunity. On the other hand, the immune evasion and pathogenicity of *K. pneumoniae* also reflect the vital role of immunity against *K. pneumoniae*. The current treatments of *hvKp* infection are controlling infection source and aggressive antibiotic therapy. However, due to the diminished effectiveness of conventional clinical treatment against antibiotic-resistant and highly pathogenic strains (Choby et al., 2020b), improving individuals’ immunity against *K. pneumoniae* infection may become a new direction for clinical therapy in the future. For the aim above, some measures, for example, regulating the gut microbiota, increasing SCFA, supplying specific antibody and so on, should be taken.

**Table 1:** Studies on the mechanism of host defense against *K. pneumoniae (Kp)* infection.

			Mechanism of host defense against *Kp*		Reference
			Mechanism	Influence	
Innate immune response	Barriers	respiratory barriers	respiratory epithelial cells	Internalize and eliminate	(Xu and Xu, 2005)
			IL-17A	Activate the defense of upper respiratory tract	(Sequeira et al., 2020)
		urinary barriers	Mechanical force of urine flow	Prevent the colonization of *Kp* on urethra	(Maunders et al., 2022)
			Urine pH↓	Affect the colonization and proliferation of bacteria	(Yang et al., 2014; Wasfi et al., 2020)
			Mechanical force of bladder contraction	Conducive to *Kp* removal	(Burnett et al., 2021)
		digestive barriers	IL-36, macrophages	Prevent the colonization and transmission of bacteria	(Sequeira et al., 2020)
			SCFA	Prevent the growth and colonization of bacteria	(Vornhagen et al., 2021)
			Combined SCFA with GPR43	Reduce the number of *Kp* and control the inflammatory response	(Galvão et al., 2018)
			Mucosal barrier	Inhibit inflammation	(Shi et al., 2017)
			Intestinal flora	Reduce liver injury	(Zheng et al., 2021)
	Innate immune cells	dendritic cells	pDCs, CD103^+^DC, MoDCs↑	Stimulate CD4^+^ and CD8^+^ naïve T cells	(Hackstein et al., 2013)
		macrophages	Release TNF-α and IL-6	Promote inflammation	(Lee et al., 2022)
			IL-36γ	Promote innate mucosal immunity in lung	(Kovach et al., 2016; Kovach et al., 2017)
			CCR2	Increase macrophages and TNF	(Chen et al., 2013)
			HIF-1α	Auxiliary the production of TNF	(Otto et al., 2021)
		neutrophils	NETs	Kill *CR-hvKP* directly	(Jin et al., 2020)
			IL-1β, IL-6, IL-17, IFN-γ, CXCL2 and TNF-α↑	Enhance ability to swallow and kill *Kp*	(Mancuso et al., 2022)
			CXCL5 and LTB_4_	Restore the activity of neutrophils	(Batra et al., 2012; Chen et al., 2016)
		Innate lymphoid cells	STAT6 signal	IL-17A↓	(Bloodworth et al., 2016)
			NK cells	Generate IFN-γ and clear *Kp*	(Ivin et al., 2017)
	Immune molecules	TRIF	recruited into the TLR complex to activates NF-κB and type I IFN	Recruit neutrophils, activate MAPKs	(Cai et al., 2009);
		MyD88	recruited into the TLR complex to activate MAPK and NF-κB signal pathways	control bacterial growth at the infection site, thus minimizing bacterial transmission	(Jeyaseelan et al., 2006; Cai et al., 2009)
		TLRs	TLR4 senses bacterial LPS	Against gram-negative bacteria	(Baral et al., 2014)
			TLR2 and TLR4 improve the levels of TNF-α, MCP-1 and iNOS	indirectly eliminate *Kp*	(Schurr et al., 2005; Jeon et al., 2017)
			TLR9 triggers the proinflammatory cascade signal	Stimulate the production of TNF-α and IL-12	(Bhan et al., 2007; Von Wulffen et al., 2007)
		NLRs	NLRP6 and NLRP12 can act as a negative regulator of the NF-κB and MAPK pathways	attenuate the intestinal inflammation	(Ghimire et al., 2020)
			NLRP12-IL-17A-CXCL1 axis	Recruit neutrophils	(Cai et al., 2016)
			IL-17 upregulate the expression of CCL2 and CCL7	Recruit neutrophils	(Xiong et al., 2015; Xiong et al., 2016)
			NLRP12 reduce the production of TNF-α and IL-6	Attenuate the inflammation caused by *Kp*	(Allen et al., 2013; Tuladhar and Kanneganti, 2020)
			NLRC4 induce the production of IL-1β and IL-17A, activating MAPK and NF-κB signaling pathways	Recruit neutrophils	(Cai et al., 2012)
Adaptive immune response	Cell-mediated immune response		Th lymphocytes secrete IL-17 and IFN-γ	plays a prominent role in the defense of *Kp*	(Lee et al., 2015)
			CD4 T_RM_ cells	Release IFN-γ or IL-4	(Amezcua Vesely et al., 2019)
			lung T_RM_ cells *via* fibroblast IL-17R signaling	provide immunity against *Kp*	(Iwanaga et al., 2021)
	Humoral immune response	MAbs	CPS antigen	Strengthen biofilm inhibition, complement deposition and NETs	(Soto et al., 2016; Diago-Navarro et al., 2017; Diago-Navarro et al., 2018; Kobayashi et al., 2018; Motley et al., 2020; Banerjee et al., 2021)
			O antigen of LPS	Promote the regulation and phagocytosis of macrophages	(Pennini et al., 2017; Berry et al., 2022)
			Vesicle antigen	Elicit EV reactive antibodies and produce IFN-γ T cell response	(Lee et al., 2015)

It is put forward that the clearance and weakening of *K. pneumoniae* colonization by host mechanical barrier can be exploited by increasing the expression of *mucin5b* gene in respiratory mucus, maintaining normal renal urinary function and avoiding dysbiosis of the intestinal flora (Roy et al., 2014). It is confirmed that an essential aspect of *Klebsiella* infection biology is the thwarting of TLR-dependent activation of host defense responses controlled by NF-κB and MAPKs (Jeyaseelan et al., 2006; Cai et al., 2009; Regueiro et al., 2011; Frank et al., 2013; Ravi Kumar et al., 2018). We hypothesize that *Klebsiella* may target the cells responsible for producing immune cytokines and blockade the signaling pathways required for the production of the cytokines. Immune cells including DCs, macrophages and neutrophils are all capable of secreting cytokines and regulating inflammatory signal pathways. Therefore, enhancing inflammatory signaling pathways such as NF-κB and MAPKs by selectively inhibiting or enhancing the secretory function of immune cells is a direction of the future research (Deets and Vance, 2021). Further, drug development and clinical trials on antibodies that can effectively bind the antigenic epitopes of *K. pneumoniae* is also a powerful anti-infection tool (Motley et al., 2020).

When it comes to the host defense, anti-*K. pneumoniae* vaccines have to be mentioned. Anti-*K. pneumoniae* vaccine based on surface-exposed bacterial antigens is a promising alternative. CPS of *K. pneumoniae* has long been regarded as a vital virulence factor that promotes resistance to phagocytosis and serum bactericidal activity. Thus, CPS has been targeted previously for the development of therapeutics and vaccines (Opoku-Temeng et al., 2019). However, the high variability in capsular serotypes limits vaccine coverage, and glycoconjugate vaccines are manufactured using intricate chemical methodologies to covalently attach purified polysaccharides to carrier proteins, which is widely considered technically challenging (Feldman et al., 2019). Joy et al. developed a preclinical model of pneumonia in mice and found that non-capsular antigens may also elicit protective immunity (Twentyman et al., 2020). As a vital virulence factor, Outer Membrane Vesicles (OMV) could induce specific adaptive immune responses while displaying intrinsic adjuvant properties. However, the side effects of the OMV vaccine, the complexity of OMV composition, and the multiple antigens in variable concentrations hinder the mass production of OMV vaccines (Martora et al., 2019). In addition, the protective efficacy of ribosome-based vaccine formulations is controversial since many include surface protein contaminations, which may be significant contributors to the protective responses (Pregliasco et al., 2009). Remarkably recombinant outer membrane proteins (OMPs) are promising vaccine candidates against *K. pneumoniae*, alone or combined with other antigens. When administered as a carrier in combination with respiratory syncytial virus subgroup A (RSV-A), OMP could induce IgA, IgG1, and IgG2a production, which provided the protection against *K. pneumoniae* infection (Libon et al., 2002). Moreover, the O antigen on LPS is a highly immunogenic molecule and an essential virulence factor for *K. pneumoniae*. However, the high toxicity of LPS is the main limiting factor related to this type of vaccine. Thus, a delicate balance between immunogenicity and toxicity must be considered (Clarke et al., 2020). Although there are no vaccines available against *K. pneumoniae* infection in clinic, it is a great pleasure that K. pneumoniae vaccines are feasible and a promising strategy to prevent infections and reduce the antimicrobial resistance burden worldwide.

In summary, there is still a considerable gap in our understanding of the pathogenesis of *K. pneumoniae*. However, in-depth knowledge of the host-immune mechanism will facilitate understanding its pathogenesis and provide new ideas for future diagnosis and treatment of *K. pneumoniae* infection in the era of antibiotics.

## Author contributions

ZL reviewed all the literature, collected data, and drafted the manuscript. YW, YL, JZ and LY drafted partly and made important suggestions for the amendments. YS conceived the review and drafted partly. XL drafted partly and reviewed the manuscript. YZ and XY contributed substantially by giving insightful comments and suggestions during the creation of the manuscript. YS, XL and XY were responsible for funding. All authors contributed to the article and approved the submitted version.

## Funding

This work was supported by grants from the National Natural Science Foundation of China (No. 81671397, 81871956, 8210072867) and Health Commission of Hubei Province Foundation (WJ2019H528).

## Conflict of interest

The authors declare that the research was conducted in the absence of any commercial or financial relationships that could be construed as a potential conflict of interest.

## Publisher’s note

All claims expressed in this article are solely those of the authors and do not necessarily represent those of their affiliated organizations, or those of the publisher, the editors and the reviewers. Any product that may be evaluated in this article, or claim that may be made by its manufacturer, is not guaranteed or endorsed by the publisher.

## References

[B1] Al Bander Z. Nitert M. D. Mousa A. Naderpoor N. (2020). The gut microbiota and inflammation: An overview. Int. J. Environ. Res. Public Health 17, 7618. doi: 10.3390/ijerph17207618 33086688 PMC7589951

[B2] Allen I. C. Mcelvania-Tekippe E. Wilson J. E. Lich J. D. Arthur J. C. Sullivan J. T. (2013). Characterization of NLRP12 during the *in vivo* host immune response to *Klebsiella pneumoniae* and *Mycobacterium tuberculosis* . PloS One 8, e60842. doi: 10.1371/journal.pone.0060842 23577168 PMC3618512

[B3] Amezcua Vesely M. C. Pallis P. Bielecki P. Low J. S. Zhao J. Harman C. C. D. (2019). Effector T_H_17 cells give rise to long-lived T cells that are essential for an immediate response against bacterial infection. Cell 178, 176–1188. doi: 10.1016/j.cell.2019.07.032 31442406 PMC7057720

[B4] Anas A. A. Claushuis T. Mohan R. A. Christoffels V. M. Aidinis V. Florquin S. (2017). Epithelial myeloid-differentiation factor 88 is dispensable during *Klebsiella pneumonia* . Am. J. Respir. Cell Mol. Biol. 56, 648–656. doi: 10.1165/rcmb.2016-0190OC 28187270

[B5] Banerjee K. Motley M. P. Diago-Navarro E. Fries B. C. (2021). Serum antibody responses against carbapenem-resistant *Klebsiella pneumoniae* in infected patients. mSphere 6, e01335-20. doi: 10.1128/mSphere.01335-20 33658281 PMC8546725

[B6] Baral P. Batra S. Zemans R. L. Downey G. P. Jeyaseelan S. (2014). Divergent functions of toll-like receptors during bacterial lung infections. Am. J. Respir. Crit. Care Med. 190, 722–732. doi: 10.1164/rccm.201406-1101PP 25033332 PMC4299612

[B7] Batra S. Cai S. Balamayooran G. Jeyaseelan S. (2012). Intrapulmonary administration of leukotriene b (4) augments neutrophil accumulation and responses in the lung to *Klebsiella* infection in CXCL1 knockout mice. J. Immunol. 188, 3458–3468. doi: 10.4049/jimmunol.1101985 22379035 PMC3311767

[B8] Bengoechea J. A. Sa Pessoa J. (2019). *Klebsiella pneumoniae* infection biology: living to counteract host defences. FEMS Microbiol. Rev. 43, 123–144. doi: 10.1093/femsre/fuy043 30452654 PMC6435446

[B9] Berry S. K. Rust S. Caceres C. Irving L. Bartholdson Scott J. Tabor D. E. (2022). Phenotypic whole-cell screening identifies a protective carbohydrate epitope on *Klebsiella pneumoniae* . MAbs 14, 2006123. doi: 10.1080/19420862.2021.2006123 34923908 PMC8726669

[B10] Bhan U. Lukacs N. W. Osterholzer J. J. Newstead M. W. Zeng X. Moore T. A. (2007). TLR9 is required for protective innate immunity in gram-negative bacterial pneumonia: role of dendritic cells. J. Immunol. 179, 3937–3946. doi: 10.4049/jimmunol.179.6.3937 17785831

[B11] Bloodworth M. H. Newcomb D. C. Dulek D. E. Stier M. T. Cephus J. Y. Zhang J. (2016). STAT6 signaling attenuates interleukin-17-producing γδ T cells during acute *Klebsiella pneumonia*e infection. Infect. Immun. 84, 1548–1555. doi: 10.1128/IAI.00646-15 26953325 PMC4862712

[B12] Bonilla F. A. Oettgen H. C. (2010). Adaptive immunity. J. Allergy Clin. Immunol. 125, S33–S40. doi: 10.1016/j.jaci.2009.09.017 20061006

[B13] Burnett L. A. Hochstedler B. R. Weldon K. Wolfe A. J. Brubaker L. (2021). Recurrent urinary tract infection: Association of clinical profiles with urobiome composition in women. Neurourol. Urodyn. 40, 1479–1489. doi: 10.1002/nau.24707 34036621 PMC8298270

[B14] Cai S. Batra S. Del Piero F. Jeyaseelan S. (2016). NLRP12 modulates host defense through IL-17A-CXCL1 axis. Mucosal. Immunol. 9, 503–514. doi: 10.1038/mi.2015.80 26349659 PMC5089371

[B15] Cai S. Batra S. Shen L. Wakamatsu N. Jeyaseelan S. (2009). Both TRIF- and MyD88-dependent signaling contribute to host defense against pulmonary *Klebsiella* infection. J. Immunol. 183, 6629–6638. doi: 10.4049/jimmunol.0901033 19846873 PMC2777750

[B16] Cai S. Batra S. Wakamatsu N. Pacher P. Jeyaseelan S. (2012). NLRC4 inflammasome-mediated production of IL-1β modulates mucosal immunity in the lung against gram-negative bacterial infection. J. Immunol. 188, 5623–5635. doi: 10.4049/jimmunol.1200195 22547706 PMC3358410

[B17] Cai S. Paudel S. Jin L. Ghimire L. Taylor C. M. Wakamatsu N.. (2021). NLRP6 modulates neutrophil homeostasis in bacterial pneumonia-derived sepsis. Mucosal. Immunol. 14, 574–584. doi: 10.1038/s41385-020-00357-4 33230225 PMC8084869

[B18] Chen K. Eddens T. Trevejo-Nunez G. Way E. E. Elsegeiny W. Ricks D. M.. (2016). IL-17 receptor signaling in the lung epithelium is required for mucosal chemokine gradients and pulmonary host defense against *K. pneumoniae* . Cell Host Microbe 20, 596–605. doi: 10.1016/j.chom.2016.10.003 27923703 PMC5149406

[B19] Chen L. Zhang Z. Barletta K. E. Burdick M. D. Mehrad B. (2013). Heterogeneity of lung mononuclear phagocytes during pneumonia: contribution of chemokine receptors. Am. J. Physiol. Lung Cell Mol. Physiol. 305, L702–L711. doi: 10.1152/ajplung.00194.2013 24056971 PMC3840272

[B20] Chew K. L. Lin R. T. P. Teo J. W. P. (2017). In Singapore: Hypervirulent infections and the carbapenemase threat. Front. Cell Infect. Microbiol. 7. doi: 10.3389/fcimb.2017.00515 PMC5732907 29312894

[B21] Choby J. E. Howard-Anderson J. Weiss D. S. (2020). Hypervirulent klebsiella pneumoniae-clinical and molecular perspectives. J. Intern. Med. 287, 283–300. doi: 10.1111/joim.13007 31677303 PMC7057273

[B22] Clarke B. R. Ovchinnikova O. G. Sweeney R. P. Kamski-Hennekam E. R. Gitalis R. Mallette E.. (2020). A bifunctional O-antigen polymerase structure reveals a new glycosyltransferase family. Nat. Chem. Biol. 16, 450–457. doi: 10.1038/s41589-020-0494-0 32152541

[B23] Deets K. A. Vance R. E. (2021). Inflammasomes and adaptive immune responses. Nat. Immunol. 22, 412–422. doi: 10.1038/s41590-021-00869-6 33603227

[B24] De Stoppelaar S. F. Claushuis T. Jansen M. P. B. Hou B. Roelofs J. J. T. H. Van’t Veer C.. (2015). The role of platelet MyD88 in host response during gram-negative sepsis. J. Thromb. Haemost. 13, 1709–1720. doi: 10.1111/jth.13048 26178922

[B25] Diago-Navarro E. Calatayud-Baselga I. Sun D. Khairallah C. Mann I. Ulacia-Hernando A.. (2017). Antibody-based immunotherapy to treat and prevent infection with *hypervirulent klebsiella pneumoniae* . Clin. Vaccine Immunol. 24, e00456-16. doi: 10.1128/cvi.00456-16 27795303 PMC5216427

[B26] Diago-Navarro E. Motley M. P. Ruiz-Peréz G. Yu W. Austin J. Seco B. M. S.. (2018). Novel, broadly reactive anticapsular antibodies against *carbapenem-resistant klebsiella pneumoniae* protect from infection. mBio 9, e00091-18. doi: 10.1128/mBio.00091-18 29615497 PMC5885035

[B27] Diekema D. J. Hsueh P.-R. Mendes R. E. Pfaller M. A. Rolston K. V. Sader H. S.. (2019). The microbiology of bloodstream infection: 20-year trends from the SENTRY antimicrobial surveillance program. Antimicrob. Agents Chemother. 63, e00355-19. doi: 10.1128/AAC.00355-19 31010862 PMC6591610

[B28] Elinav E. Strowig T. Kau A. L. Henao-Mejia J. Thaiss C. A. Booth C. J.. (2011). NLRP6 inflammasome regulates colonic microbial ecology and risk for colitis. Cell 145, 745–757. doi: 10.1016/j.cell.2011.04.022 21565393 PMC3140910

[B29] Feldman M. F. Mayer Bridwell A. E. Scott N. E. Vinogradov E. Mckee S. R. Chavez S. M.. (2019). A promising bioconjugate vaccine against *hypervirulent klebsiella pneumoniae* . Proc. Natl. Acad. Sci. U. S. A. 116, 18655–18663. doi: 10.1073/pnas.1907833116 31455739 PMC6744904

[B30] Frank C. G. Reguerio V. Rother M. Moranta D. Maeurer A. P. Garmendia J.. (2013). *Klebsiella pneumoniae* targets an EGF receptor-dependent pathway to subvert inflammation. Cell Microbiol. 15, 1212–1233. doi: 10.1111/cmi.12110 23347154

[B31] Galvão I. Tavares L. P. Corrêa R. O. Fachi J. L. Rocha V. M. Rungue M.. (2018). The metabolic sensor GPR43 receptor plays a role in the control of *Klebsiella pneumoniae* infection in the lung. Front. Immunol. 9. doi: 10.3389/fimmu.2018.00142 PMC5826235 29515566

[B32] Ghimire L. Paudel S. Jin L. Jeyaseelan S. (2020). The NLRP6 inflammasome in health and disease. Mucosal. Immunol. 13, 388–398. doi: 10.1038/s41385-020-0256-z 31988468 PMC7493825

[B33] Hackstein H. Kranz S. Lippitsch A. Wachtendorf A. Kershaw O. Gruber A. D.. (2013). Modulation of respiratory dendritic cells during *Klebsiella pneumonia* infection. Respir. Res. 14, 91. doi: 10.1186/1465-9921-14-91 24044871 PMC3848864

[B34] Hackstein H. Wachtendorf A. Kranz S. Lohmeyer J. Bein G. Baal N. (2012). Heterogeneity of respiratory dendritic cell subsets and lymphocyte populations in inbred mouse strains. Respir. Res. 13, 94. doi: 10.1186/1465-9921-13-94 23066782 PMC3493345

[B35] Holt K. E. Wertheim H. Zadoks R. N. Baker S. Whitehouse C. A. Dance D.. (2015). Genomic analysis of diversity, population structure, virulence, and antimicrobial resistance in *Klebsiella pneumoniae*, an urgent threat to public health. Proc. Natl. Acad. Sci. U. S. A. 112, e3574–e3581. doi: 10.1073/pnas.1501049112 26100894 PMC4500264

[B36] Ivin M. Dumigan A. De Vasconcelos F. N. Ebner F. Borroni M. Kavirayani A.. (2017). Natural killer cell-intrinsic type I IFN signaling controls *Klebsiella pneumoniae* growth during lung infection. PloS Pathog. 13, e1006696. doi: 10.1371/journal.ppat.1006696 29112952 PMC5675380

[B37] Iwanaga N. Chen K. Yang H. Lu S. Hoffmann J. P. Wanek A.. (2021). Vaccine-driven lung T_RM_ cells provide immunity against *Klebsiella via* fibroblast IL-17R signaling. Sci. Immunol. 6, eabf1198. doi: 10.1126/sciimmunol.abf1198 34516780 PMC8796208

[B38] Jagnow J. Clegg S. (2003). *Klebsiella pneumoniae* MrkD-mediated biofilm formation on extracellular matrix- and collagen-coated surfaces. Microbiol. (Reading) 149, 2397–2405. doi: 10.1099/mic.0.26434-0 12949165

[B39] Jeon H.-Y. Park J.-H. Park J.-I. Kim J.-Y. Seo S.-M. Ham S.-H.. (2017). Cooperative interactions between toll-like receptor 2 and toll-like receptor 4 in murine *Klebsiella pneumoniae* infections. J. Microbiol. Biotechnol. 27, 1529–1538. doi: 10.4014/jmb.1704.04039 28595383

[B40] Jeyaseelan S. Young S. K. Yamamoto M. Arndt P. G. Akira S. Kolls J. K.. (2006). Toll/IL-1R domain-containing adaptor protein (TIRAP) is a critical mediator of antibacterial defense in the lung against *Klebsiella pneumoniae* but not *Pseudomonas aeruginosa.* J. Immunol 177, 538–547. doi: 10.4049/jimmunol.177.1.538 16785551

[B41] Jin L. Liu Y. Jing C. Wang R. Wang Q. Wang H.. (2020). Neutrophil extracellular traps (NETs)-mediated killing of *carbapenem-resistant hypervirulent* (*CR-hvKP*) are impaired in patients with diabetes mellitus. Virulence 11, 1122–1130. doi: 10.1080/21505594.2020.1809325 32865110 PMC7549946

[B42] Kobayashi S. D. Porter A. R. Freedman B. Pandey R. Chen L. Kreiswirth B. N.. (2018). Antibody-mediated killing of carbapenem-resistant ST258 *Klebsiella pneumoniae* by human neutrophils. mBio 9, e00297-18. doi: 10.1128/mBio.00297-18 29535199 PMC5850326

[B43] Kovach M. A. Singer B. Martinez-Colon G. Newstead M. W. Zeng X. Mancuso P.. (2017). IL-36γ is a crucial proximal component of protective type-1-mediated lung mucosal immunity in gram-positive and -negative bacterial pneumonia. Mucosal. Immunol. 10, 1320–1334. doi: 10.1038/mi.2016.130 28176791 PMC5548659

[B44] Kovach M. A. Singer B. H. Newstead M. W. Zeng X. Moore T. A. White E. S.. (2016). IL-36γ is secreted in microparticles and exosomes by lung macrophages in response to bacteria and bacterial components. J. Leukoc. Biol. 100, 413–421. doi: 10.1189/jlb.4A0315-087R 26864267 PMC4945350

[B45] Lee W. H. Choi H. I. Hong S. W. Kim K. S. Gho Y. S. Jeon S. G.. (2015). Vaccination with *Klebsiella pneumoniae*-derived extracellular vesicles protects against bacteria-induced lethality *via* both humoral and cellular immunity. Exp. Mol. Med. 47, e183. doi: 10.1038/emm.2015.59 26358222 PMC4650931

[B46] Lee I. M. Huang T.-Y. Yang F.-L. Johansson V. Hsu C.-R. Hsieh P.-F.. (2022). A hexasaccharide from capsular polysaccharide of *carbapenem-resistant klebsiella pneumoniae KN2* is a ligand of toll-like receptor 4. Carbohydr. Polym. 278, 118944. doi: 10.1016/j.carbpol.2021.118944 34973762

[B47] Libon C. Haeuw J. F. Crouzet F. Mugnier C. Bonnefoy J. Y. Beck A.. (2002). *Streptococcus pneumoniae* polysaccharides conjugated to the outer membrane protein a from *Klebsiella pneumoniae* elicit protective antibodies. Vaccine 20, 2174–2180. doi: 10.1016/s0264-410x(02)00149-4 12009270

[B48] Li Y. Han W. Y. Li Z. J. Lei L. C. (2009). *Klebsiella pneumoniae* MrkD adhesin-mediated immunity to respiratory infection and mapping the antigenic epitope by phage display library. Microb. Pathog. 46, 144–149. doi: 10.1016/j.micpath.2008.11.006 19100827

[B49] Li W. Hu Y. Zhang Q. Hua L. Yang Z. Ren Z.. (2021). Development of drug-resistant vaccine *via* novel vesicle production technology. ACS Appl. Mater. Interfaces 13, 32703–32715. doi: 10.1021/acsami.1c06701 34251169

[B50] Li B. Zhao Y. Liu C. Chen Z. Zhou D. (2014). Molecular pathogenesis of *Klebsiella pneumoniae* . Future Microbiol. 9, 1071–1081. doi: 10.2217/fmb.14.48 25340836

[B51] Mackel J. J. Morffy Smith C. Wasbotten R. K. Twentyman J. Rosen D. A. (2022). Classical and γδ T cells are each independently sufficient to establish protection against a classical strain of. Klebsiella pneumoniae. Front. Cell Infect. Microbiol. 12. doi: 10.3389/fcimb.2022.974175 PMC9471189 36118033

[B52] Mamantopoulos M. Ronchi F. Van Hauwermeiren F. Vieira-Silva S. Yilmaz B. Martens L.. (2017). *Nlrp6-* and ASC-dependent inflammasomes do not shape the commensal gut microbiota composition. Immunity 47, 339–348.e334. doi: 10.1016/j.immuni.2017.07.011 28801232

[B53] Mancuso P. Curtis J. L. Weitzel A. M. Griffin C. A. Bouchard B. Freeman C. M.. (2022). Diet-induced obesity in mice impairs host defense against pneumonia *in vivo* and glucose transport and bactericidal functions in neutrophils *in vitro* . Am. J. Physiol. Lung Cell Mol. Physiol. 322, L116–L128. doi: 10.1152/ajplung.00008.2021 34850640 PMC8794018

[B54] Martora F. Pinto F. Folliero V. Cammarota M. Dell'annunziata F. Squillaci G.. (2019). Isolation, characterization and analysis of pro-inflammatory potential of *Klebsiella pneumoniae* outer membrane vesicles. Microb. Pathog. 136, 103719. doi: 10.1016/j.micpath.2019.103719 31493501

[B55] Maunders E. A. Ganio K. Hayes A. J. Neville S. L. Davies M. R. Strugnell R. A.. (2022). The role of ZntA in *Klebsiella pneumoniae* zinc homeostasis. Microbiol. Spectr. 10, e0177321. doi: 10.1128/spectrum.01773-21 35019689 PMC8754117

[B56] Motley M. P. Diago-Navarro E. Banerjee K. Inzerillo S. Fries B. C. Fries B. C. (2020). The role of IgG subclass in antibody-mediated protection against *carbapenem-resistant klebsiella pneumoniae* . mBio 11, e02059-20. doi: 10.1128/mBio.02059-20 32900809 PMC7482069

[B57] Myers J. A. Miller J. S. (2021). Exploring the NK cell platform for cancer immunotherapy. Nat. Rev. Clin. Oncol. 18, 85–100. doi: 10.1038/s41571-020-0426-7 32934330 PMC8316981

[B58] Na L. Bo F. Wang G. X. Shi X. F. Zheng S. Ren L.. (2014). Studies affecting the role of *Klebsiella pneumoniae* in adherence to epithelial cells. Sichuan J. Physiol. Sci. 36, 3–6.

[B59] O’neill L. Golenbock D. Bowie A. G. (2013). The history of toll-like receptors-redefining innate immunity. Nat. Rev. Immunol. 13, 453–460. doi: 10.1038/nri3446 23681101

[B60] Opoku-Temeng C. Kobayashi S. D. Deleo F. R. (2019). *Klebsiella pneumoniae* capsule polysaccharide as a target for therapeutics and vaccines. Comput. Struct. Biotechnol. J. 17, 1360–1366. doi: 10.1016/j.csbj.2019.09.011 31762959 PMC6861629

[B61] Opoku-Temeng C. Malachowa N. Kobayashi S. D. Deleo F. R. (2022). Innate host defense against *Klebsiella pneumoniae* and the outlook for development of immunotherapies. J. Innate Immun. 14, 167–181. doi: 10.1159/000518679 34628410 PMC9149408

[B62] Otto N. A. Pereverzeva L. Leopold V. Ramirez-Moral I. Roelofs J. J. T. H. Van Heijst J. W. J.. (2021). Hypoxia-inducible factor-1 in macrophages, but not in neutrophils, is important for host defense during-induced pneumosepsis. Mediators. Inflamm. 2021, 9958281. doi: 10.1155/2021/9958281 34393650 PMC8360744

[B63] Paczosa M. K. Mecsas J. (2016). *Klebsiella pneumoniae*: Going on the offense with a strong defense. Microbiol. Mol. Biol. Rev. 80, 629–661. doi: 10.1128/MMBR.00078-15 27307579 PMC4981674

[B64] Pallmer K. Oxenius A. (2016). Recognition and regulation of T cells by NK cells. Front. Immunol. 7. doi: 10.3389/fimmu.2016.00251 PMC4919350 27446081

[B65] Pennini M. E. De Marco A. Pelletier M. Bonnell J. Cvitkovic R. Beltramello M.. (2017). Immune stealth-driven O2 serotype prevalence and potential for therapeutic antibodies against multidrug resistant *Klebsiella pneumoniae* . Nat. Commun. 8, 1991. doi: 10.1038/s41467-017-02223-7 29222409 PMC5722860

[B66] Pichavant M. Delneste Y. Jeannin P. Fourneau C. Brichet A. Gosset P.. (2003). Outer membrane protein a from *Klebsiella pneumoniae* activates bronchial epithelial cells: implication in neutrophil recruitment. J. Immuno. 171, 6697–6705. doi: 10.4049/jimmunol.171.12.6697 14662873

[B67] Plantinga M. Guilliams M. Vanheerswynghels M. Deswarte K. Branco-Madeira Toussaint W.. (2013). Conventional and monocyte-derived CD11b(+) dendritic cells initiate and maintain T helper 2 cell-mediated immunity to house dust mite allergen. Immunity 38, 322–335. doi: 10.1016/j.immuni.2012.10.016 23352232

[B68] Pregliasco F. Terracciano L. Marcassa S. Zava D. Anselmi G. (2009). Rationale for the clinical use of a ribosome-component immune modulator. Allergy Asthma Proc. 30 Suppl 1, S5–12. doi: 10.2500/aap.2009.30.3249 19679000

[B69] Ramirez-Moral I. Blok D. C. Bernink J. H. Garcia-Laorden M. I. Florquin S. Boon L.. (2021). Interleukin-33 improves local immunity during gram-negative pneumonia by a combined effect on neutrophils and inflammatory monocytes. J. Pathol. 253, 374–383. doi: 10.1002/path.5601 33305354 PMC7986604

[B70] Ravi Kumar S. Paudel S. Ghimire L. Bergeron S. Cai S. Zemans R. L.. (2018). Emerging roles of inflammasomes in acute pneumonia. Am. J. Respir. Crit. Care Med. 197, 160–171. doi: 10.1164/rccm.201707-1391PP 28930487 PMC5768907

[B71] Regueiro V. Moranta D. Frank C. G. Larrarte E. Margareto J. March C.. (2011). *Klebsiella pneumoniae* subverts the activation of inflammatory responses in a NOD1-dependent manner. Cell Microbiol. 13, 135–153. doi: 10.1111/j.1462-5822.2010.01526 20846183

[B72] Rodríguez-Medina N. Barrios-Camacho H. Duran-Bedolla J. Garza-Ramos U. (2019). *Klebsiella variicola*: an emerging pathogen in humans. Emerg. Microbes Infect. 8, 973–988. doi: 10.1080/22221751.2019.1634981 31259664 PMC6609320

[B73] Roy M. G. Livraghi-Butrico A. Fletcher A. A. Mcelwee M. M. Evans S. E. Boerner R. M.. (2014). *Muc5b* is required for airway defence. Nature 505, 412–416. doi: 10.1038/nature12807 24317696 PMC4001806

[B74] Russo T. A. Marr C. M. (2019). Hypervirulent klebsiella pneumoniae. Clin. Microbiol. Rev. 32, e00001-19. doi: 10.1128/CMR.00001-19 31092506 PMC6589860

[B75] Saharman Y. R. Karuniawati A. Sedono R. Aditianingsih D. Goessens W. H. F. Klaassen C. H. W.. (2020). Clinical impact of endemic NDM-producing *Klebsiella pneumoniae* in intensive care units of the national referral hospital in Jakarta, Indonesia. Antimicrob. Resist. Infect. Control 9, 61. doi: 10.1186/s13756-020-00716-7 32393386 PMC7216366

[B76] Schurr J. R. Young E. Byrne P. Steele C. Shellito J. E. Shellito J. E.. (2005). Central role of toll-like receptor 4 signaling and host defense in experimental pneumonia caused by gram-negative bacteria. Infect. Immun. 73, 532–545. doi: 10.1128/IAI.73.1.532-545.2005 15618193 PMC538991

[B77] Sequeira R. P. Mcdonald J. Marchesi J. R. Clarke T. B. (2020). Commensal bacteroidetes protect against *Klebsiella pneumoniae* colonization and transmission through IL-36 signalling. Nat. Microbiol. 5, 304–313. doi: 10.1038/s41564-019-0640-1 31907407 PMC7610889

[B78] Shi N. Li N. Duan X. Niu H. (2017). Interaction between the gut microbiome and mucosal immune system. Mil. Med. Res. 4, 14. doi: 10.1186/s40779-017-0122-9 28465831 PMC5408367

[B79] Soto E. Marchi S. Beierschmitt A. Kearney M. Francis S. VanNess K.. (2016). Interaction of non-human primate complement and antibodies with *hypermucoviscous klebsiella pneumoniae* . Vet. Res. 47, 40. doi: 10.1186/s13567-016-0325-1 26951091 PMC4782414

[B80] Tuladhar S. Kanneganti T.-D. (2020). NLRP12 in innate immunity and inflammation. Mol. Aspects Med. 76, 100887. doi: 10.1016/j.mam.2020.100887 32838963 PMC9375713

[B81] Twentyman J. Morffy Smith C. Nims J. S. Dahler A. A. Rosen D. A. Rosen D.A. (2020). A murine model demonstrates capsule-independent adaptive immune protection in survivors of *Klebsiella pneumoniae* respiratory tract infection. Dis. Model. Mech. 13, dmm043240. doi: 10.1242/dmm.043240 32298236 PMC7104859

[B82] Von Wulffen W. Steinmueller M. Herold S. Marsh L. M. Bulau P. Seeger W.. (2007). Lung dendritic cells elicited by fms-like tyrosine 3-kinase ligand amplify the lung inflammatory response to lipopolysaccharide. Am. J. Respir. Crit. Care Med. 176, 892–901. doi: 10.1186/1465-9921-14-91 17690334

[B83] Vornhagen J. Bassis C. M. Ramakrishnan S. Hein R. Mason S. Bergman Y.. (2021). A plasmid locus associated with *Klebsiella* clinical infections encodes a microbiome-dependent gut fitness factor. PloS Pathog. 17, e1009537. doi: 10.1371/journal.ppat.1009537 33930099 PMC8115787

[B84] Wang G. Zhao G. Chao X. Xie L. Wang H. Wang H. (2020). The characteristic of virulence, biofilm and antibiotic resistance of *Klebsiella pneumoniae* . Int. J. Environ. Res. Public Health 17, 6278. doi: 10.3390/ijerph17176278 32872324 PMC7503635

[B85] Wasfi R. Abdellatif G. R. Elshishtawy H. M. Ashour H. M. (2020). First-time characterization of viable but non-culturable proteus mirabilis: Induction and resuscitation. J. Cell Mol. Med. 24, 2791–2801. doi: 10.1111/jcmm.15031 32030883 PMC7077546

[B86] Wolff N. S. Jacobs M. C. Wiersinga W. J. Hugenholtz F. (2021). Pulmonary and intestinal microbiota dynamics during gram-negative pneumonia-derived sepsis. Intensive Care Med. Exp. 9, 35. doi: 10.1186/s40635-021-00398-4 34250564 PMC8272965

[B87] Wu T. Xu F. Su C. Li H. Lv N. Liu Y.. (2020). Alterations in the gut microbiome and cecal metabolome during *Klebsiella pneumoniae*-induced pneumosepsis. Front. Immunol. 11. doi: 10.3389/fimmu.2020.01331 PMC7411141 32849494

[B88] Xiong H. Carter R. A. Leiner I. M. Tang Y.-W. Chen L. Kreiswirth B. N.. (2015). Distinct contributions of neutrophils and CCR2^+^ monocytes to pulmonary clearance of different *Klebsiella pneumoniae* strains. Infect. Immun. 83, 3418–3427. doi: 10.1128/IAI.00678-15 26056382 PMC4534658

[B89] Xiong H. Keith J. W. Samilo D. W. Carter R. A. Leiner I. M. Pamer E. G.. (2016). Innate Lymphocyte/Ly6C(hi) monocyte crosstalk promotes *Klebsiella pneumoniae* clearance. Cell 165, 679–689. doi: 10.1016/j.cell.2016.03.017 27040495 PMC4842125

[B90] Xu X. Weiss I. D. Zhang H. H. Singh S. P. Wynn T. A. Wilson M. S.. (2014). Conventional NK cells can produce IL-22 and promote host defense in *Klebsiella pneumoniae* pneumonia. J. Immunol. 192, 1778–1786. doi: 10.4049/jimmunol.1300039 24442439 PMC3995347

[B91] Xu Y. Xu H. (2005). Small airway epithelial cell injury induced by *Klebsiella pneumoniae* in rats and its relationship with intrapulmonary alpha tumor necrosis factor. Chin. J. Biochem. Drugs 1, 12–14. doi: 10.3969/j.issn.1005-1678.2005.01.0012-03

[B92] Yang L. Wang K. Li. H. Denstedt J. D. Cadieux P. A. (2014). The influence of urinary pH on antibiotic efficacy against bacterial uropathogens. Urology 84, 731.e731–737. doi: 10.1016/j.urology.2014.04.048 25168568

[B93] Zheng D. Cao R. Lin X. Wang J. (2014). Effect of airborne PM2.5 exposure on the inflammatory response to *Klebsiella pneumoniae* in rat lungs. Basic Med. Clin. 34, 1110–1112. doi: 10.16352/j.issn.1001-6325.2014.08.017

[B94] Zheng Y. Ding Y. Xu M. Chen H. Zhang H. Liu Y.. (2021). Gut microbiota contributes to host defense against *Klebsiella pneumoniae*-induced liver abscess. J. Inflamm. Res. 14, 5215–5225. doi: 10.2147/jir.S334581 34675599 PMC8519413

